# Blood Serum from Patients with Acute Leukemia Inhibits the Growth of Bone Marrow Multipotent Mesenchymal Stromal Cells

**DOI:** 10.3390/biomedicines13051265

**Published:** 2025-05-21

**Authors:** Nataliya Petinati, Aleksandra Sadovskaya, Irina Shipounova, Alena Dorofeeva, Nina Drize, Anastasia Vasilyeva, Olga Aleshina, Olga Pokrovskaya, Larisa Kuzmina, Sofia Starchenko, Valeria Surimova, Yulia Chabaeva, Sergey Kulikov, Elena Parovichnikova

**Affiliations:** National Medical Research Center for Hematology, Moscow 125167, Russia; sadovskaya.a@blood.ru (A.S.); shipunova.i@blood.ru (I.S.); dorofeeva.a@blood.ru (A.D.); vasilieva.a@blood.ru (A.V.); gavrilina.o@blood.ru (O.A.); pokrovskaya.o@blood.ru (O.P.); kuzmina.l@blood.ru (L.K.); starchenko.s@blood.ru (S.S.); surimova.v@blood.ru (V.S.); chabaeva.y@blood.ru (Y.C.); kulikov.s@blood.ru (S.K.); parovichnikova.e@blood.ru (E.P.)

**Keywords:** multipotent mesenchymal stromal cells, blood serum, acute leukemia, allogeneic hematopoietic stem cell transplantation

## Abstract

**Background/Objectives**: Acute leukemia (AL) alters both hematopoiesis and the bone marrow stromal microenvironment. Attempts to develop a culture of multipotent mesenchymal stromal cells (MSCs) from AL patients’ bone marrow are not always successful, as opposed to healthy donors’ bone marrow. **Methods**: To unveil the reason, healthy donors’ MSCs were cultured in the presence of sera from healthy donors (control group) or AL patients at the onset of the disease, in short- and long-term remission, and before and after allogeneic hematopoietic stem cell transplantation (allo-HSCT). **Results**: The cell yield in the presence of patient sera was lower than in the control, regardless of the AL stage. It was assumed that the patients either lacked growth factors to sustain MSCs, or there were inhibitors of MSC growth present. The serum’s ability to support MSC growth correlated with platelet count and albumin and calcium concentrations in patients’ blood. Platelet-derived growth factors—PDGFA and PDGFB—are known to induce MSC growth. Their concentration in the serum of AL patients and healthy donors was analyzed. A decrease in PDGFA concentration was found in the sera of patients compared to healthy donors. PDGFB concentration was lower at disease onset, increased during remission and decreased again during relapse. PDGFB concentration correlated with platelet count, while PDGFA concentration did not. AL patients’ sera reflected systemic disturbances affecting MSC growth. So far, decreases in PDGFs, albumin and calcium concentration, as well as platelet count, are the parameters that might be among the causes of this observation.

## 1. Introduction

Acute leukemia (AL) is a clonal oncological blood disease. The proliferation of malignant cells disrupts the body’s normal functions. Normal hematopoiesis is suppressed, and the secretion of hormones, growth factors and cytokines into the blood is altered [1]. Understanding the changes in the blood and bone marrow can provide insights into the etiology and prognosis of AL. Circulating proteins can serve as markers for diagnosis, treatment selection, or risk stratification. Recent studies have revealed drastic shifts in the blood serum proteome composition associated with several diseases, including leukemia [2,3,4]. In leukemia, changes in the interactions between serum proteins and their environment can affect both the malignant cells and the bone marrow stroma [5]. This may contribute to damage of the hematopoietic niches of the bone marrow stroma. In AL patients, the stroma never completely reverts to the state of healthy donors, even after bone marrow transplantation and long-term remission [6].

Multipotent mesenchymal stromal cells (MSCs) from bone marrow play a leading role in organizing a niche for hematopoietic stem cells, thus regulating hematopoiesis [7,8]. In leukemia, MSCs can be recruited by the malignant cells to remodel the niche, suppress normal hematopoiesis and promote tumor cells’ survival [9]. MSCs can protect tumor cells from chemotherapy. Preventing the interaction of MSCs with tumor cells can improve the response to therapy. For example, according to preliminary data, the inhibition of Aurora kinase prevents MSCs from supporting tumor stem cells [10]. Inhibiting MSC Wnt signaling allows for drug resistance to be overcome [11]. Intraosteously introduced healthy donor MSCs can support hematopoiesis after allogeneic hematopoietic stem cell transplantation (allo-HSCT) [12].

Typically, MSCs are relatively easy to isolate and expand in culture. This property is crucial for the development and clinical applications of MSC-based therapies. We have consistently succeeded in culturing MSCs from the bone marrow of healthy donors. However, in the case of some AL patients, our efforts were unsuccessful despite using the same procedure and conditions. One potential explanation is the systemic changes caused by leukemia. It is known that the growth of MSCs in vitro depends on the serum added to the medium. Disturbances affecting the growth of MSCs in the bone marrow can be reflected in the blood sera obtained from patients. When comparing newly diagnosed patients with acute myeloid leukemia (AML) and acute lymphoblastic leukemia (ALL), AML showed a significant increase in IL-4, IL-2 and IL-3, and a significant decrease in VEGF and VCAM-1 [13].

Many factors influence MSC growth. PDGFB [14] and FGF2 [15] stimulate their proliferation. TGFB, BMP-2, BMP-3, EGF, HGF and VEGF support their survival and differentiation [16]. PDGFA regulates the chemotaxis [17], migration and trophic function of MSCs [18].

Patients’ sera might lack the necessary growth factors or contain substances that negatively affect bone marrow stromal progenitor cells. A hypothesis was put forward that the sera of AL patients could adversely impact the MSCs of healthy donors.

The aim of the study was to investigate the effect of the blood sera of AL patients at the onset of the disease and at different stages of therapy on the MSCs of healthy donors.

## 2. Materials and Methods

Blood sera from healthy donors and patients with AL was obtained when collecting blood for clinical tests. MSCs from healthy donors and patients were isolated from 3 to 5 mL of bone marrow obtained during exfusion from healthy donors and diagnostic puncture from patients after they signed informed consent, as described previously [19]. Sera from 141 patients with AL (72 with AML and 69 with ALL) were analyzed before treatment and after achieving remission, and 36 serum samples from patients before and after allo-HSCT (20 with AML and 17 with ALL). Serum from 16 healthy donors was used as a control. The serum was stored at −40 °C.

The biological samples were obtained, if possible, from the same patients at different stages of treatment.

MSCs were cultured in αMEM medium (Hyclone, Logan, UT, USA) with 10% fetal calf serum (Hyclone, USA), 2 mM glutamine (Hyclone, USA), 100 U/mL penicillin (Sintez, Moscow, Russia) and 50 μg/mL streptomycin (BioPharmGarant, Vladimir, Russia) at 37 °C and 5% CO_2_, as described previously [20]. Nine independent experiments were carried out on MSCs from 6 healthy donors.

If there was no growth of fibroblast-like cell colonies in 30 days after bone marrow cell seeding, it was assumed that the sample did not contain MSCs able to proliferate in culture. Such cultures were labeled as “no MSC growth”.

To analyze cell growth and proliferation, the MTT test (CellTiter 96AQ, Promega, Madison, WI, USA) was performed according to the manufacturer’s recommendations. Donor MSCs were cultured in a 96-well plate at 1000 cells per well in αMEM medium (Hyclone, USA) with 2 mM glutamine (Hyclone, USA), 100 U/mL penicillin (Sintez, Russia), 50 μg/mL streptomycin (BioPharmGarant, Russia) and either 10% fetal calf serum (Hyclone, USA) or 10% serum of healthy donors or AL patients at the onset, remission, relapse or long-term remission (>2 years), before or after allo-HSCT at 37 °C and 5% CO_2_ for a week. To calculate the cell count per well based on the optical density (OD) value in the MTT assay, a calibration curve was constructed. Based on the calibration, the cell count was determined using the formula: cell count = 7768.5 × OD − 342.08 (Figure S1, Supplementary Materials).

For cytokine and chemokine content analysis in sera, a part of the Bio-Plex Pro Human Cytokine Panel, 27-Plex (BioRad, Hercules, CA, USA) was used to determine the concentrations of IL-7, IL-8, IL-10, IL-13, G-CSF, GM-CSF, MCP-1 (MCAF), MIP-1β and TNF-α according to the manufacturer’s recommendations. Detection of fluorescent beads was performed on a Bio-Plex 200 system (BioRad, USA). All measurements were made in triplicate.

Enzyme-linked immunosorbent assay kits (Cloud-Clone Corp, Wuhan, China) were used to determine FGF2, PDGFA and PDGFB content according to the manufacturer’s recommendations. The sera of 7 patients at the onset and in remission of AML (2 of them in relapse), and 6 patients at the onset and in remission of ALL (2 of them in relapse) were analyzed.

Hemograms and biochemical analyses were performed as part of routine clinical care. Since patients had their blood tests taken multiple times during treatment, the data on the day closest (within 5 days) to the date of serum sample collection were analyzed (Table S2, Supplementary Materials).

Statistical analysis was performed using GraphPad Prism version 8.1 (GraphPad Software Inc., Boston, MA, USA) and SAS V9.4. For a simple comparison of means in the independent samples with normal distribution, Student’s *t*-test was used; in the case of abnormal data distribution, the Mann–Whitney U-test was used for unpaired comparisons, and the Wilcoxon test for paired comparisons. Then, for some calculations, OD data were normalized (divided) by the mean OD value of the control group separately for each experiment to account for differences in the proliferative potential of MSCs from different healthy donors. Then, the OD values were logarithmized (base 10) to normalize their distribution and allow for their analysis using linear models. The data contained internal correlation due to the repetitive measurements from the same subjects. Therefore, methods of variance regression analysis on repeated observations were used (MIXED procedure of the SAS V9.4 package) [21].

## 3. Results

**Figure 1:**
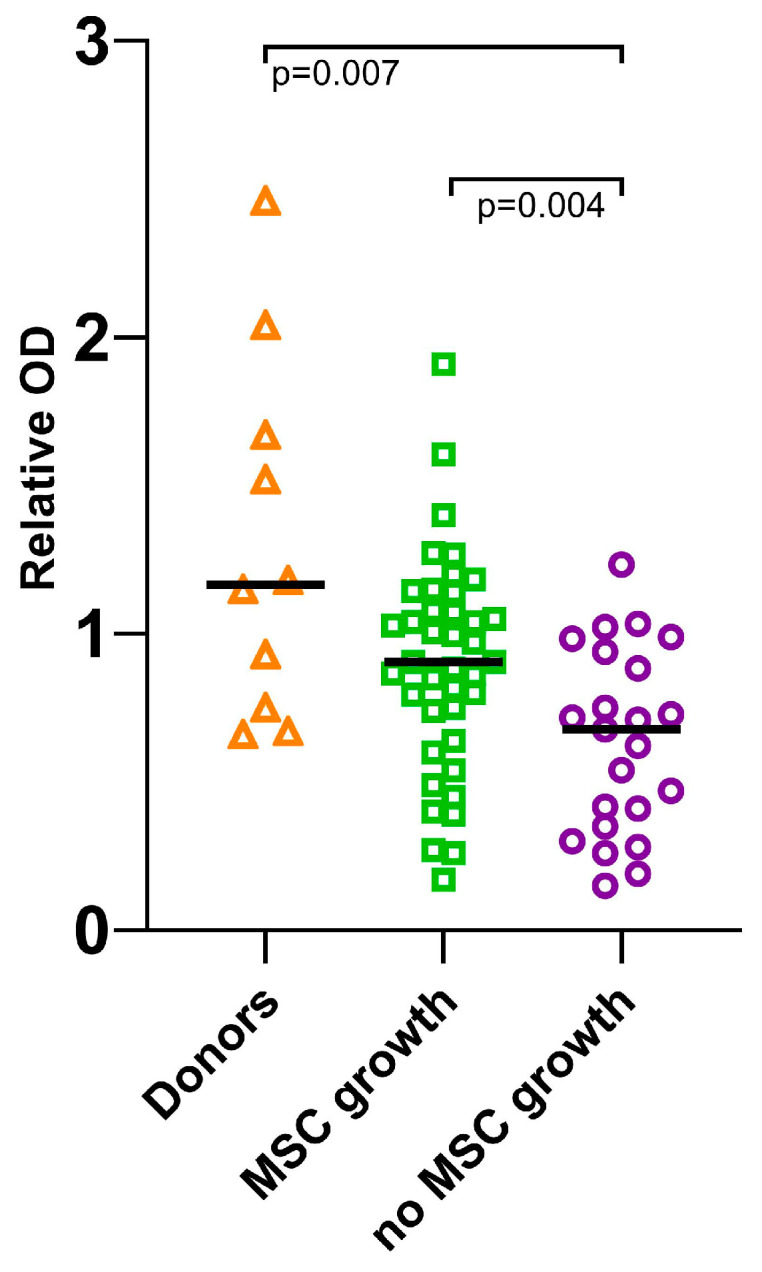
Optical density (OD) of donor MSCs cultured in the presence of healthy donors’ or AL patients’ sera, depending on whether or not the patient’s MSCs grew. The scatter plot shows relative OD measurements in three groups, left to right: culture with healthy donor sera; culture with the sera of the patients whose MSCs grew; culture with the sera of the patients whose MSCs did not grow; the line denotes the median. The significance was calculated using the Mann–Whitney U-test.

The study began with an investigation of sera from the patients whose MSCs did not grow at the onset of the disease. It was suggested that the bone marrow stromal precursors of these patients were damaged. Impaired growth of the stromal precursors in patients could be caused not only by local changes in the bone marrow, but also by external signals transmitted through the blood. In the latter case, the patients’ blood sera could either contain substances that inhibited MSC growth, or lack substances necessary for MSC survival and growth. To test this, MSCs from healthy donors were cultured in the presence of the patients’ sera for a week. It was found that MSCs from healthy donors grew significantly worse in the presence of patients’ sera compared to the sera of healthy donors (median OD = 1.17). Moreover, the sera of patients whose MSCs did not grow in 30 days (median OD = 0.68) sustained the MSCs much worse compared to the sera of patients from whom it was possible to develop MSC cultures (median OD = 0.91) (Figure 1).

Thus, the hypothesis about blood-borne signals was confirmed. The serum concentration of cytokines affecting MSC growth was analyzed. In the sera of AL patients whose MSCs grew and did not grow in culture, trends towards differences with the sera of healthy donors were revealed (Table 1); however, the differences were not significant.

In the patients’ sera, the average concentrations of interleukins IL8 (2-fold), IL10 (5-9-fold), IL13 (3-fold) and MIP-1b were increased compared to healthy donors, but there were no statistically significant differences between the groups.

**Figure 2:**
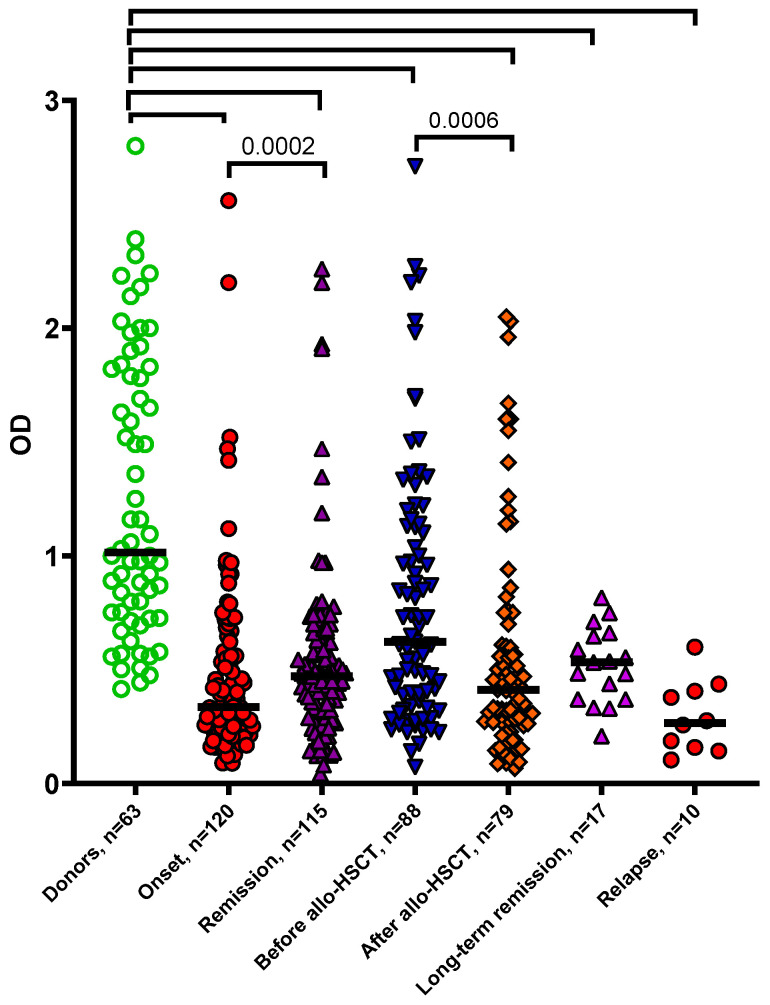
Effect of sera from patients with acute leukemia at different stages of disease on the growth of MSCs from healthy donors.

The study was set up to investigate whether the patient sera obtained at different stages of treatment affected the MSCs of healthy donors differentially. Non-normalized data on optical density in the MTT test of the MSCs of healthy donors cultured in the presence of healthy donors’ sera and the sera of AL patients at all stages of treatment are presented in Figure 2 and Table 2.

The optical density (OD) data shown are not normalized. The data are presented as a scatter plot; the line denotes the median. Statistical analysis was performed using the Mann–Whitney U-test. The *p*-value of the noted pairs was <0.0001 if not indicated otherwise.

It is noteworthy that even with long-term remission of more than 2 years, the sera of patients supported the growth of donor MSCs worse than the sera of healthy donors. Therefore, the blood serum did not restore its potential to sustain bone marrow stromal cells after allo-HSCT nor during long-term remission.

The next aim was to study the dynamics of the patient serum properties at different stages of therapy (Figure S2A, Supplementary Materials) and whether it depended on the diagnosis (ALL or AML) (Figure S2B, Supplementary Materials). Both hypotheses were tested with the analysis of variance on repeated measurements. The analysis showed that the serum samples taken at the onset were different in their MSC-sustaining ability from the sera obtained in remission and before allo-HSCT. After allo-HSCT or in relapse, the ability of the serum to support MSC growth dropped again and was no different from the onset group (Table S2, Supplementary Materials). The analysis showed no statistically significant differences between ALL and AML (Table S3, Supplementary Materials).

Obviously, either the sera of AL patients contain an inhibitor of MSC growth, or the concentration of factors necessary to maintain the normal growth of these cells is reduced, or both.

Some of the main known MSC growth factors are platelet-derived growth factors—PDGFA and PDGFB—and FGF2. The concentration of these proteins in the blood sera of healthy donors and patients with AL was studied with the ELISA method. A significant decrease in the concentration of PDGFA and PDGFB was found in the sera of patients compared to the sera of healthy donors, whereas the concentration of FGF2 at the onset and remission of the disease was higher than in healthy donors (Table 3). No difference in the concentration of PDGFA between the onset, remission and relapse was found. The concentration of PDGFB was reduced at the onset of AL compared to healthy donors, increased in remission and decreased in relapse. The concentration of these factors in the blood serum did not depend on AL subtype.

These data indicate that the concentration of PDGFs was reduced in the blood sera of patients with AL at the onset and remission of the disease. Since these are growth factors secreted by platelets, platelet levels in the blood of patients and healthy donors were analyzed at the time of serum sampling. A correlation was observed between the platelet count and PDGFB concentration (Table 3). PDGFA and FGF2 concentration did not depend on the platelet count at the time of the study. In remission, FGF2 concentration correlated inversely with OD (Pearson r = −0.713, *p* = 0.009).

In addition, biochemical blood parameters and complete blood count with leukocyte count on the day of the serum sampling were analyzed. Hematological and biochemical blood parameters of patients changed significantly during treatment, so the dynamics of several parameters were analyzed (Tables S4 and S5, Supplementary Materials).

In the studied cohort of AL patients, the leukocyte counts, blast cells, platelets and lymphocytes at the onset of the disease were expectedly significantly different from those in the healthy donor group. Calcium and albumin indices, although within normal values in most patients, were also significantly different from the healthy donor group (Tables S4 and S5, Figure S3, Supplementary Materials). Upon achieving remission, the indices normalized, but did not reach the average values of the healthy donor group and, with the exception of albumin and calcium, continued to differ significantly from them both before and after allo-HSCT.

The correlation of the sera’s ability to sustain donor MSC growth and the blood parameters was analyzed. A link was found between the OD values and the platelet level at all studied stages of the disease. The highest correlation coefficients were observed at the onset (Table 4). It can be assumed that the platelets, which supply numerous growth factors to the blood, are an important promoter of stromal precursor growth, albeit not the only one.

A correlation of OD with the platelet count and the concentration of calcium and albumin in the blood was revealed in variance regression analysis on repeated observations. It turned out that of all the parameters studied, albumin and calcium had the greatest correlation with sustaining MSC growth. Changes in their concentrations (within physiological limits) correlated with normalized OD values (Table S6, Supplementary Materials). Both the levels of calcium and albumin changed significantly with the stages of treatment. The most notable difference in both calcium and albumin levels was observed between the blood samples of the patients before and after allo-HSCT. The dynamics of calcium and albumin seemed to mimic the OD dynamics to some extent. Therefore, the next stage of the analysis was an attempt to adjust the OD values for the values of platelet count, calcium and albumin. In order to do that, the values of these factors were imported into the regression model constructed for OD dynamics (Figure S3, Supplementary Materials). The adjusted OD dynamics were noticeably “smoothed out”; the statistical significance of both the model itself and the individual differences between the stages disappeared. This suggests that the biochemical composition of the serum affects MSC proliferation and explains the observed OD dynamics to some extent. No significant difference in the blood composition nor effect of sera on the growth of donor MSCs was observed between AML and ALL patients.

## 4. Discussion

When establishing cultures of AL patients’ MSCs, we found out that in some cases, the cells did not grow—unlike healthy donors’, from whose bone marrow we were always able to develop MSC cultures. We already knew that MSCs were significantly altered in acute leukemia compared to the MSCs of a healthy donor [6,22]. We assumed that the patient’s internal environment created conditions unfavorable for MSCs, and, as a result, the cells were not always capable of growth even after being transferred to culture. We studied the blood serum, as it often reflects systemic changes in the body. It turned out that the sera of patients at all stages of AL had impaired ability to support the growth of MSCs from healthy donors.

The search for tumor transformation markers and outcome predictors in the blood sera of patients has been underway for many years. It has been shown that of the three components of the CONUT nutritional status assessment—serum albumin, serum cholesterol and absolute lymphocyte count in the blood—only the lymphocyte count is associated with the prognosis in cancer patients [23]. However, a high CONUT score is an unfavorable prognostic sign in myelodysplastic syndrome and AML. Another unfavorable prognostic sign in AL patients may be an increase in serum endocan, a proteoglycan secreted by endothelial cells. It is associated with early non-relapse mortality after allo-HSCT [24].

Analysis of serum protein composition is another promising research area. Zheng and colleagues found changes in the sera of AL patients compared to the sera of healthy donors: decreased levels of α1-trypsin inhibitor, trypsin inhibitor, prealbumin and apolipoproteins E and A-IV, as well as increased levels of retinol-binding protein, globin HP2, serum lectin, factor H protein homolog and A1 amyloid [25]. In addition, increased levels of cobalamin (vitamin B12) [26], angiogenin [27], IL-2 receptor [28], lactate dehydrogenase and heavy metal ions [29] were found in AL patients’ sera. In the sera of children with acute promyelocytic leukemia, 33 proteins were elevated and 50 proteins were decreased compared to the sera of healthy donors. S100A8 and LRG1 can be noted among the elevated proteins, and SPARC among the decreased [30].

In a study analyzing the protein content of AML patients’ sera, statistically significant changes in the content of physiologically important proteins were revealed compared to the sera of healthy donors [3]. Most of these proteins were secreted by MSCs among other cells. We compared several proteins that were found both in the sera and in MSCs’ secretomes of AL patients and healthy donors, based on Jajula et al.’s study and our previous study (Table 5).

As shown, proteins that presented differentially in serum proteomes, for the most part, did not differ significantly in the MSC secretomes. It can be assumed that the contribution of MSCs to the protein composition of serum is very low, and these cells do not impact the changes seen in the patients’ sera.

The functional properties of serum can be impacted not only by the quantity of proteins, but also by their activity. For example, calcineurin activity in the sera of patients with ALL and AML decreased by 25% and 15%, respectively, compared to the activity in the sera of healthy donors. After treatment, calcineurin activity was restored in AML, but not in ALL. At the same time, the concentration of calcineurin, like calmodulin, did not differ between the groups [32].

Unfortunately, all these data do not allow us to evaluate the factors directly related to the growth of healthy donor MSCs in the presence of patients’ sera. In this study, we analyzed the parameters of patients’ and healthy donors’ blood taken at the same time as the samples of serum added to the MSC media. Sera from AL patients had an impaired ability to support the growth of MSCs from healthy donors. This may be due to a lack of growth factors necessary for the bone marrow stroma or to the presence of inhibitors secreted by tumor cells. The content of albumin and total calcium in the blood correlated with the ability of the serum to support MSC growth. Albumin is added to semi-liquid media for the growth of progenitor cells [33] and is necessary for the in vitro analysis of extracellular vesicles [34] due to its ability to bind lipids. Low serum albumin levels correlated with poor maintenance of healthy donor MSC growth. Along with albumin, the concentration of Ca^2+^ in the sera of patients decreased. However, its concentration did not go beyond normal values. Calcium maintains homeostasis and is necessary for a large number of cellular processes, including responses to many signals. Its low serum levels might lead to functional insufficiency and a slower or weaker response to stimuli. Traditionally, calcium-dependent signaling was considered to control platelet activation. Recent advances indicate that both extracellular and intracellular Ca^2+^ fluxes, mediated by membrane transporters (extracellular flux) and endoplasmic reticulum–mitochondria coupling (intracellular flux), contribute to platelet activation [35]. As a secondary messenger, calcium flux regulates cytoskeletal reorganization; granule, microvesicle and exosome release; platelet aggregation; and thrombus formation [36]. Upon degranulation, platelets release the contents of their granules, including growth and coagulation factors, chemoattractants (from α-granules) and ATP, serotonin and calcium (from δ-granules) [37,38]. Thus, there might be a link between platelet number, platelet activity and serum Ca^2+^ concentration.

A decrease in the concentration of PDGFA and PDGFB, necessary for MSC proliferation, in the blood sera of patients indicated a lack of growth factors in the sera of patients. These factors are produced by many tissues [39]. Receptors for them are present on MSCs. PDGF-BB along with FGF2, LIF and EGF are the main regulators of MSC proliferation [40]. Platelets release PDGFs along with TGFb, FGF2, IGF1, VEGF and EGF and other factors [41].

Under the influence of patients’ sera, the MSCs’ proliferative activity in culture decreased. It has been shown that in the presence of FGF2 and PDGFB, MSCs were not only induced to proliferate, but also activate, while platelet lysate did not induce MSC activation [42]. Since the level of PDGFB was associated with the platelet count, it is understandable why the PDGFB level increased along with the restoration of the platelet count in remission, in contrast to the concentration of PDGFA. The concentration of PDGFA was reduced in the sera of patients at all stages of treatment and did not correlate with the platelet count. The levels of PDGFA and PDGFB did not correlate with OD; however, this may have been due to the much smaller cohort of patients for whom these cytokines’ levels were measured. It can be assumed that the PDGF deficiency in the sera of patients affected the ability of their sera to support the growth of MSCs from healthy donors. It is obvious that PDGFA and PDGFB are only a small part of the factors secreted by platelets and involved in the maintenance and proliferation of MSCs.

The number of platelets in the blood of patients was reduced, which might have also meant a decrease in TGFb, LIF, EGF and other factors secreted by platelets. It is known that MSCs grow better in the presence of platelet-rich plasma [43,44], which supports the hypothesis of a lack of growth factors in the sera of patients. FGF2 is also secreted by platelets, but its concentration was increased in the patients’ sera, implying an additional source of it. It seems that an increase in FGF2 concentration did not improve the serum’s capacity to sustain MSCs; in fact, the opposite trend was observed in AL remission.

The presence of inhibitors in the sera of AL patients can be inferred from our in vitro observations. In some cases, patients’ MSCs fail to grow in culture. The sera from such patients were less efficient in supporting the growth of healthy donor MSCs than the sera from patients whose MSCs were successfully cultured. Even the sera of patients in long-term remission did not achieve the same level of MSC support as healthy donors’, despite complete restoration of their platelet counts. Further research is needed to understand why recovery is incomplete and to develop methods to maintain stromal precursors in patients who are in remission. Such methods could potentially benefit patients undergoing allo-HSCT, as our data shows that their sera had a very low level of MSC support. Moreover, restoring the stroma could enhance the engraftment.

## 5. Conclusions

The lack of factors supporting the growth of bone marrow stromal progenitor cells in the blood sera of AL patients was revealed. These factors may have a systemic effect on the patient. Thus, leukemia affects not only hematopoiesis, but also stromal progenitors of bone marrow.

## Figures and Tables

**Table 1:** Cytokine concentrations (pg/mL) in the sera of healthy donors and AL patients. The data are presented as mean values ± standard deviation. Differences between the groups in all parameters are statistically insignificant.

	Healthy Donors (*n* = 11)	Patients Whose MSCs Grew (*n* = 11)	Patients Whose MSCs Did Not Grow (*n* = 10)
MIP-1b	20.89 ± 8.40	27.85 ± 4.48	25.63 ± 3.23
TNF	32.80 ± 0.01	28.76 ± 4.92	38.93 ± 12.75
IL-13	1.55 ± 0.01	3.95 ± 0.30	3.71 ± 0.38
MCP-1	25.07 ± 5.16	18.60 ± 7.29	27.62 ± 9.74
IL-8	25.05 ± 17.37	53.31 ± 17.02	50.17 ± 14.60
IL-10	1.37 ± 1.11	5.73 ± 2.09	11.83 ± 3.88
G-CSF	32.83 ± 0.01	13.29 ± 7.98	31.51 ± 10.32
IL-7	18.02 ± 4.06	20.59 ± 5.76	21.31 ± 5.99

**Table 2:** Cell production of donor MSCs cultured in the presence of healthy donor sera and sera from AML and ALL patients at different stages of the disease.

Sera			Cells Per Well	*p*-Value ▲
Healthy donors	(*n* = 9)		5079 ± 529	
Patients	ALL (*n* = 26)	Onset	2283 ± 230	<0.001
	AML (*n* = 47)		1977 ± 189 *	<0.001
	ALL (*n* = 21)	Remission	2565 ± 308	<0.001
	AML (*n* = 40)		2955 ± 221 *	0.001
	ALL (*n* = 16)	Before allo-HSCT	3262 ± 535 **	0.023
	AML (*n* = 24)		3058 ± 370	0.006
	ALL (*n* = 15)	After allo-HSCT	1770 ± 229 **	<0.001
	AML(*n* = 20)		1899 ± 254	<0.001
	ALL (*n* = 5)	Relapse	2136 ± 618	0.012
	AML(*n* = 7)		1666 ± 371	<0.001
	AML, ALL, APL (*n* = 17)	Long-term remission	3685 ± 313	<0.001

▲ *p*-value of the difference with the healthy donor group; * significant difference between AML onset and remission, *p* = 0.004; ** significant difference between ALL patients before and after allo-HSCT, *p* = 0.001.

**Table 3:** Concentration of PDGFs and FGF2 in the blood sera of healthy donors and AL patients at different stages of the disease.

	PDGFA		PDFGB			FGF2	
	Concentration, pg/mL (M ± SE)	Difference with Healthy Donors, *p*-Value	Concentration, pg/mL (M ± SE)	Difference with Healthy Donors, *p*-Value	Correlation with Platelet Count	Concentration, pg/mL (M ± SE)	Difference with Healthy Donors, *p*-Value
Healthy donors (*n* = 10)	1239 ± 100		6.0 ± 1.43		Spearman r = 0.346, *p* = 0.33	244.6 ± 23.4	
Onset (*n* = 13)	831 ± 122	*p* = 0.0173	0.41 ± 0.27	*p* = 0.0035	Spearman r = 0.578, *p* = 0.038	375.3 ± 32.62	*p* = 0.0058
Remission (*n* = 13)	824 ± 117	*p* = 0.0134	3.21 ± 0.97	*p* = 0.1264	Pearson r = 0.684, *p* = 0.01	322 ± 25.47	*p* = 0.0415
Relapse (*n* = 4)	846 ± 228	*p* = 0.18556	0.14 ± 0.09	*p* = 0.0027	Pearson r = 0.959, *p* = 0.04	280.2 ± 28.05	*p* = 0.4069

**Table 4:** Correlation coefficients of the biochemical blood composition and complete blood count with the OD of MSCs from healthy donors cultured in the presence of AL patient sera.

Diagnosis	Stage	Leukocytes	Platelets	Lymphocytes	Albumin	Calcium Total
ALL	onset	0.038	0.560	0.052	0.075	0.161
	remission	0.137	0.311	0.281	0.081	−0.181
	before allo-HSCT	0.095	0.712	−0.246	0.336	0.300
	after allo-HSCT	0.349	0.435	0.280	−0.049	0.166
AML	onset	0.009	0.580	0.000	0.320	0.325
	remission	0.325	0.381	0.293	0.251	0.040
	before allo-HSCT	0.102	0.266	0.133	0.497	0.423
	after allo-HSCT	0.197	0.383	0.386	−0.309	0.281

**Table 5:** Proteins presented differentially in the sera of AML patients compared to healthy donors, identified by LFQ analysis [3], and the same proteins presented in the secretomes of MSCs from AML patients compared to MSCs from healthy donors, identified using mass spectrometry [31].

Protein Name	Blood Serum [3]		MSC Secretome [31]	
	Log2-Fold Change	*p*-Value	Log2-Fold Change	*p*-Value
ANXA1	6.83	0.008		
Peroxiredoxin-5, mitochondrial PRDX5	5.6	0.04	0.005	0.16
ARPC5	5.53	0.02	−0.013/inf	0.27/0.008
ARPC4	4.38	0.02	−0.669	0.88
PTGDS	4.22	0.02	−0.41	0.57
RAC1	−4.36	0.02	−3.36	0.2
COL1A1	−4.9	0.03	−0.109	0.04
IDH2 mitochondrial	−5.21	0.001		
PGM1	−5.29	0.02	−1.13	0.5
HSPD1 mitochondrial	−5.83	0.02	−1.75	0.06
HNRNPK	−6.37	0.000085	−0.41	0.15
CTSC	−6.43	0.03	−1.22	0.54

## Data Availability

The data presented in this study are available from the corresponding author on reasonable request.

## Supplementary Materials

The following supporting information can be downloaded at: https://www.mdpi.com/article/10.3390/biomedicines13051265/s1, Figure S1: Calibration curve for calculation of cell number based on optical density in MTT test; Figure S2: Analysis of variance of optical density (OD) data obtained in the MTT test of donor MSCs cultured in the presence of sera of donors and patients at different AL stages; Figure S3: Results of the analysis of changes in OD at different stages of therapy without correction and after correction for the values of platelets, calcium and albumin in the blood (305 observations); Table S1: Characteristics of sera and corresponding blood indices; Table S2: Results of the analysis of changes in OD at different stages of therapy (558 observations); Table S3: Results of the analysis of changes in OD at different stages of therapy (558 observations); Table S4: Data on OD, indicators of biochemical blood composition and hemogram at different stages of therapy; Table S5: Statistical significance of differences in parameters between pairs of groups in the Mann-Whitney test; Table S6: Correlations of blood indices and normalized OD.

## Abbreviations

The following abbreviations are used in this manuscript:

AL	Acute Leukemia
ALL	Acute Lymphoblastic Leukemia
Allo-HSCT	Allogeneic Hematopoietic Stem Cell Transplantation
AML	Acute Myeloid Leukemia
MSCs	Mesenchymal Stromal Cells
OD	Optic Density
