# Supplementary material for: Blood Serum from Patients with Acute Leukemia Inhibits the Growth of Bone Marrow Multipotent Mesenchymal Stromal Cells
Source: Biomedicines. 2025 May 21;13(5):1265. doi: 10.3390/biomedicines13051265 (PMC12109124; doi:10.3390/biomedicines13051265)
Supplement: Supplementary file 1 [file biomedicines-13-01265-s001.zip › Supplement english.pdf]

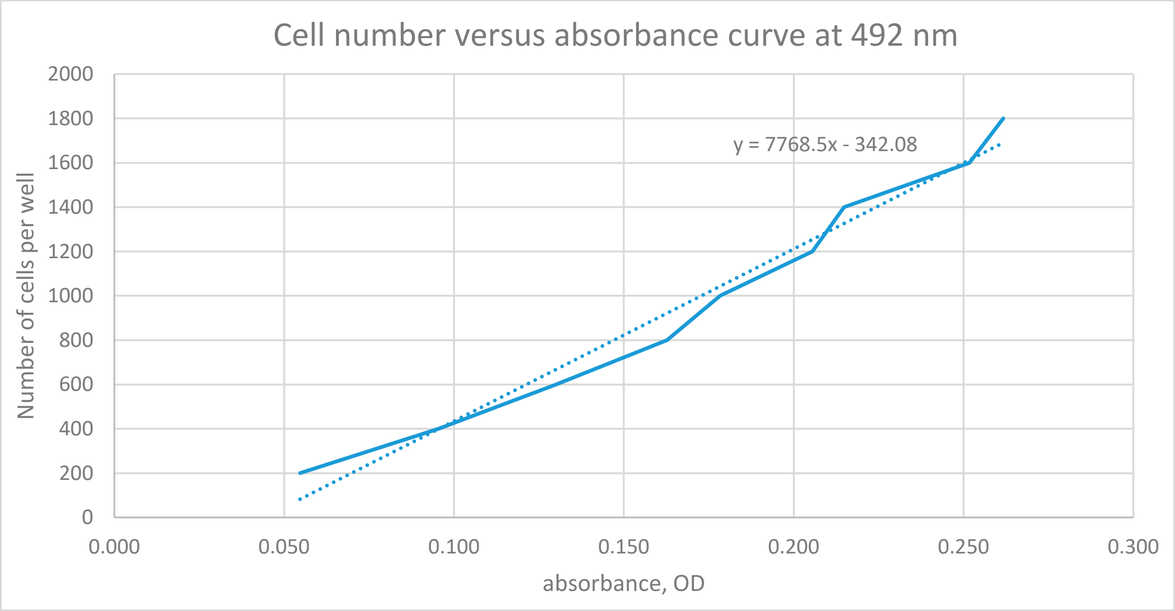

Figure S1. Calibration curve for calculation of cell number based on optical density in MTT test.

Table S1. Characteristics of sera and corresponding blood indices. – see in separate Excel file.

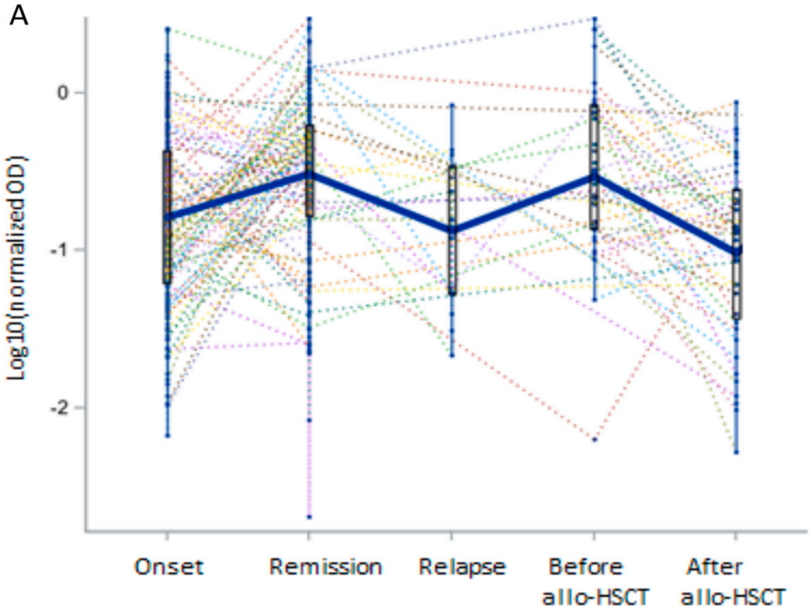

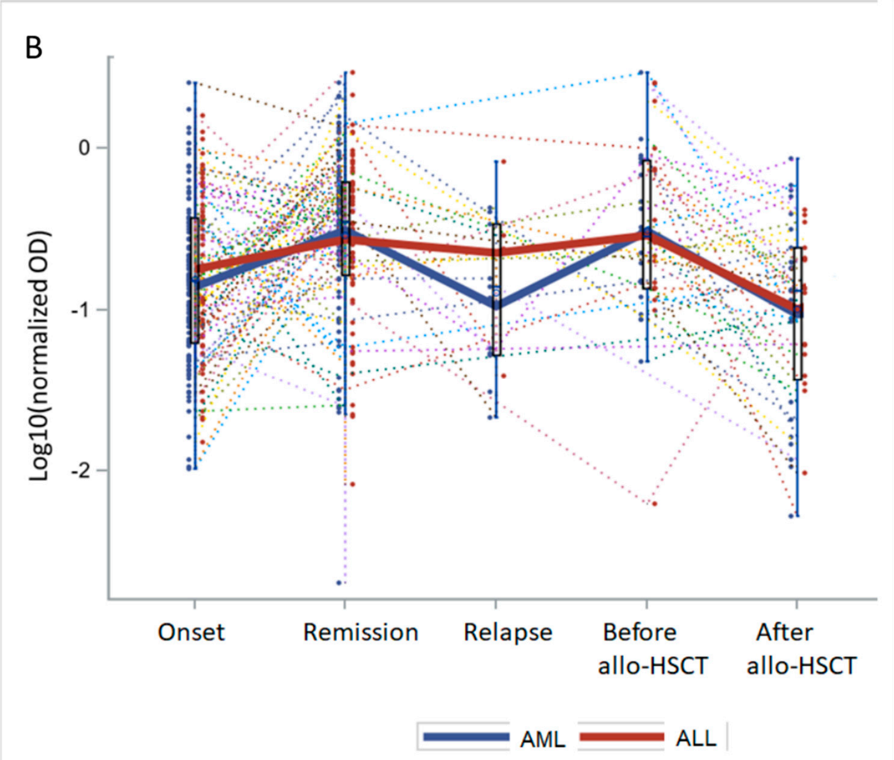

Figure S2. Analysis of variance of optical density (OD) data obtained in the MTT test of donor MSCs cultured in the presence of sera of donors and patients at different AL stages.

The data are normalized to donor values and logarithmized. The average optical density value obtained during MSC growth on donor sera is taken as 0. Adjusted mean (bold line) values and scatter characteristics (box plots) of OD values of patients at different stages of therapy (at AL onset, remission, relapse, before allo-HSCT (remission for at least 3 months) and after allo-HSCT).

A. All patients.

B. Trend lines for AML and ALL patients shown separately.

Table S2. Results of the analysis of changes in OD at different stages of therapy (558 observations). Variance-regression model on repeated measurements.

Stage of therapy	Adjusted mean (lsmeans) of log OD for patients in the AL group	Significance of the difference (Pr > |t|) from the reference level	General significance of the model
Onset	-0.26	reference	
Remission	-0.16	<.0001	
Relapse	-0.26	0.99	
Before allo-HSCT	-0.13	<.0001	0.004
After allo-HSCT	-0.28	0.39	

Table S3. Results of the analysis of changes in OD at different stages of therapy (558 observations). Variance-regression model on repeated measurements. Comparison of ALL and AML groups.

Stage of therapy	Adjusted mean (lsmeans) of log OD for patients in the ALL versus AML group	Significance of the difference (Pr > |t|) from the reference level	General significance of the model
Onset	-0.17	reference	
Remission	-0.14	0.55	
Relapse	-0.30	0.09	
Before allo-HSCT	-0.17	0.95	0.40
After allo-HSCT	-0.25	0.14	

Table S4. Data on OD, indicators of biochemical blood composition and hemogram at different stages of therapy.

ALL	OD in relation to donors (M±SE)	Leukocytes, 10^9/L	Platelets*, 10^9/L	Lymphocytes, 10^9/L	Albumin**, g/L	Calcium total***, mM
onset n=62	0.537±0.03	51.71±10.48	99.23±9.63	4.19±0.60	40.10±0.60	2.31±0.02
remission n=58	0.67±0.04	3.33±0.33	219.27±19.72	1.03±0.09	41.13±0.58	2.31±0.02
before allo-HSCT n=29	0.748±0.07	4.103±0.35	202.467±17.44	1.441±0.20	43.430±0.83	2.356±0.03
after allo-HSCT n= 36	0.468±0.04	2.722±0.33	118.171±13.12	1.008±0.18	40.005±0.80	2.302±0.02
**AML**						
onset n=105	0.68±0.03	47.00±6.70	57.89±6.32	37.08±0.52	39.52±0.53	2.52±0.15
remission n=98	0.68±0.03	3.70±0.25	199.30±12.32	0.88±0.06	40.09±0.44	2.35±0.01
before allo-HSCT n=44	0.72±0.04	3.31±0.28	184.16±13.42	1.05±0.04	43.18±0.56	2.390±.12
after allo-HSCT n=44	0.56±0.04	2.88±0.25	124.40±11.00	0.88±0.15	51.99±9.98	2.310±0.02

*Reference value for adults (164-369)x10^9/L.

**Reference value for adults 35-52 g/L.

***Reference value for adults 2.20-2.65 mM.

Table S5. Statistical significance of differences in parameters between pairs of groups in the Mann-Whitney test.

	**OD in relation to donors**	**Leukocytes**	**Platelets**	**Lymphocytes**	**Albumin**	**Calcium total**
Donors versus AL onset	<0.001	0,0368	<0.001	0,4673	<0.001	<0.001
Donors versus AL remission	<0.001	<0.001	0,0270	0,0029	0,0053	0,0078
Donors versus before allo-HSCT	<0.001	<0.001	0,0140	0,0067	0,8131	0,0931
Donors versus after allo-HSCT	<0.001	<0.001	<0.001	0,0759	0,0003	0,0014
AL onset versus AL remission	<0.001	<0.001	<0.001	<0.001	0,0151	0,0012
before allo-HSCT versus after HSCT	<0.001	0,0314	<0.001	0,0090	0,0002	0,0159
ALL onset versus ALL remission	0.009	<0.001	<0.001	0,0049	0,1264	0,7123
ALL patients before allo-HSCT versus after allo-HSCT	<0.001	0,0106	0,0009	0,4043	0,0733	0,1140
AML onset versus AML remission	<0.001	<0.001	<0.001	<0.001	0,0373	0,0002
AML patients before allo-HSCT versus after allo-HSCT	0.004	0,7256	0,0090	0,0045	0,0011	0,0764

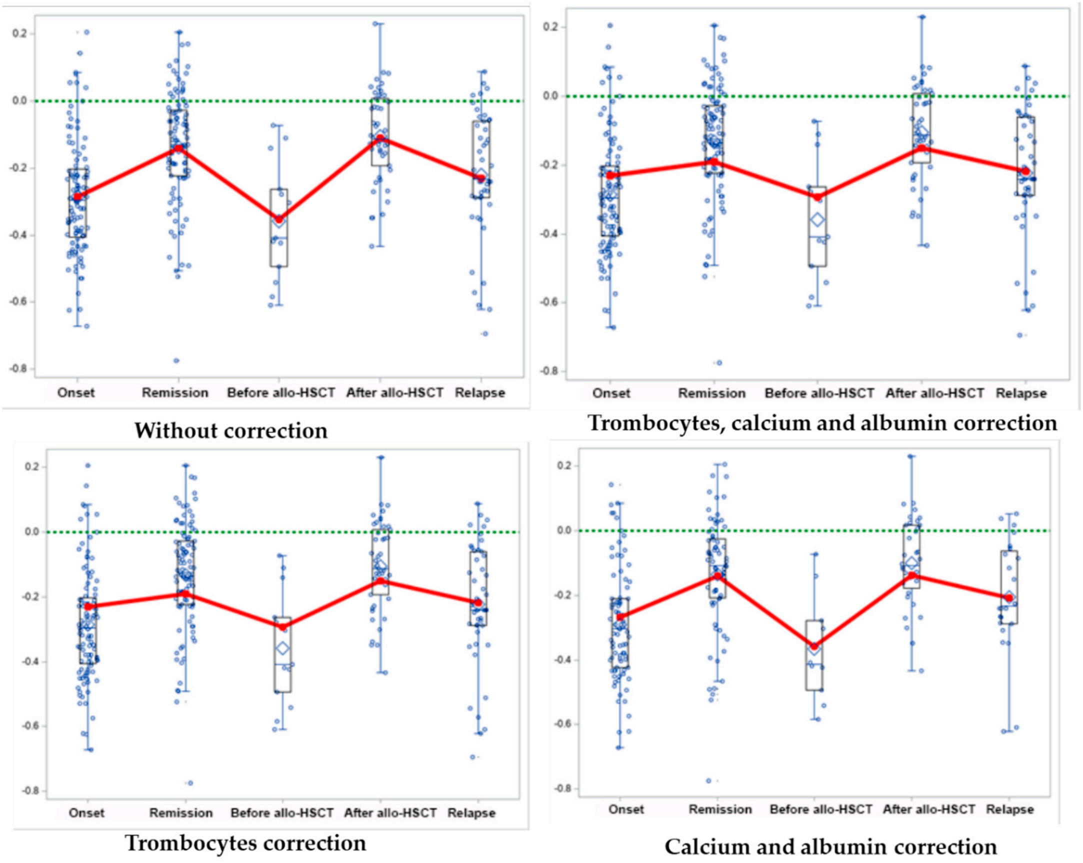

Figure S3. Results of the analysis of changes in OD at different stages of therapy without correction and after correction for the values of platelets, calcium and albumin in the blood (305 observations). Variance-regression model on repeated measurements. Zero on the ordinate axis corresponds to the average OD value in the presence of donor sera.

Table S6. Correlations of blood indices and normalized OD.

Blood index	Correlation coefficient	P-value
Total calcium	0.46	<0.0001
Albumin	0.36	<0.0001
Uric acid	0.30	<0.0001
Direct bilirubin	0.14	<0.0001
Total granulocyte count	0.28	<0.0001
Myelocytes	0.23	<0.0001
Segmented nucleobases	0.28	<0.0001
Blast cells	-0.20	<0.0002
